# Case report: detection of multiple sporadic gastrointestinal stromal tumors by dual-time ^18^ F-FDG PET/CT

**DOI:** 10.3389/fonc.2024.1321179

**Published:** 2024-03-28

**Authors:** Chuan Li, Wenxin Li, Maocai Shang, Pan Wang, Xianwen Hu

**Affiliations:** Affiliated Hospital of Zunyi Medical University, Department of Nuclear Medicine, Zunyi, China

**Keywords:** 18 F-FDG, PET/CT, gastrointestinal stromal tumor, abdomen, GISTs

## Abstract

Gastrointestinal stromal tumors (GISTs) are the most common mesenchymal tumors affecting the gastrointestinal tract. Typically, GISTs are solitary; however, in rare cases, they may be multiple and appear in one or more organs. Multiple GISTs can appear in familial GISTs, children, or certain tumor syndromes such as neurofibromatosis type 1, Carney syndrome, and Carney-Stratakis syndrome. However, the diagnosis of primary multiple sporadic GISTs is often more difficult than that of these diseases. Herein, we report a case of multiple primary sporadic GISTs in a 64-year-old man, affecting the abdominal cavity and retroperitoneum, as identified through dual-time point positron emission tomography (PET) with ^18^F-labeled fluoro-2-deoxyglucose (^18^F-FDG) and computed tomography (^18^F-FDG PET/CT). Notably, the dual-time-point PET/CT revealed the migration of masses near the lower abdomen into the abdominal cavity. Furthermore, a significant increase in radioactive uptake of the mass 3 h after ^18^F-FDG injection compared with that 1 h after injection may be an important cue for its diagnosis.

## 1 Introduction

Gastrointestinal stromal tumors (GISTs) are typically solitary, with rare instances of multiple occurrences (1). Existing literature has primarily associated the occurrence of multiple GISTs with specific scenarios, including individuals under 30 years of age (2), familial GISTs (hereditary GISTs) (3), neurofibromatosis type 1 (NF1) (4), and Carney’s triad and Carney-Stratakis syndrome (5, 6). Pediatric GISTs and the Carney triad and Carney-Stratakis syndrome, in particular, have frequently presented as multiple GISTs (2, 5, 6), while NFl and familial GIST syndrome often involve numerous tumors in one or more parts of the digestive tract, accompanied by hyperplasia of the diffuse interstitial cells of Cajal. The small intestine is a common site for NFl, while familial GISTs often manifest as multiple primary tumors at multiple sites (3, 4). These diseases exhibit unique clinical characteristics owing to their specific pathogenesis and medical history, facilitating clinical diagnosis. Primary multiple sporadic GISTs are rare and have only recently been confirmed (7, 8). These multiple primary tumors can simultaneously appear in the same or different parts of the same patient (stomach, intestine, or peritoneum), each displaying different KIT or PDGFRA mutation types (9). Herein, we report a case of primary multiple sporadic GISTs revealed by dual-time point positron emission tomography (PET) with ^18^F-labeled fluoro-2-deoxyglucose (^18^F-FDG) and computed tomography (^18^F-FDG PET/CT), with the purpose of exploring the diagnostic value of dual time point PET/CT in GIST and enhancing the understanding of this rare disease, providing valuable insights for both imaging diagnostic and clinical doctors.

## 2 Case description

**Figure 1:**
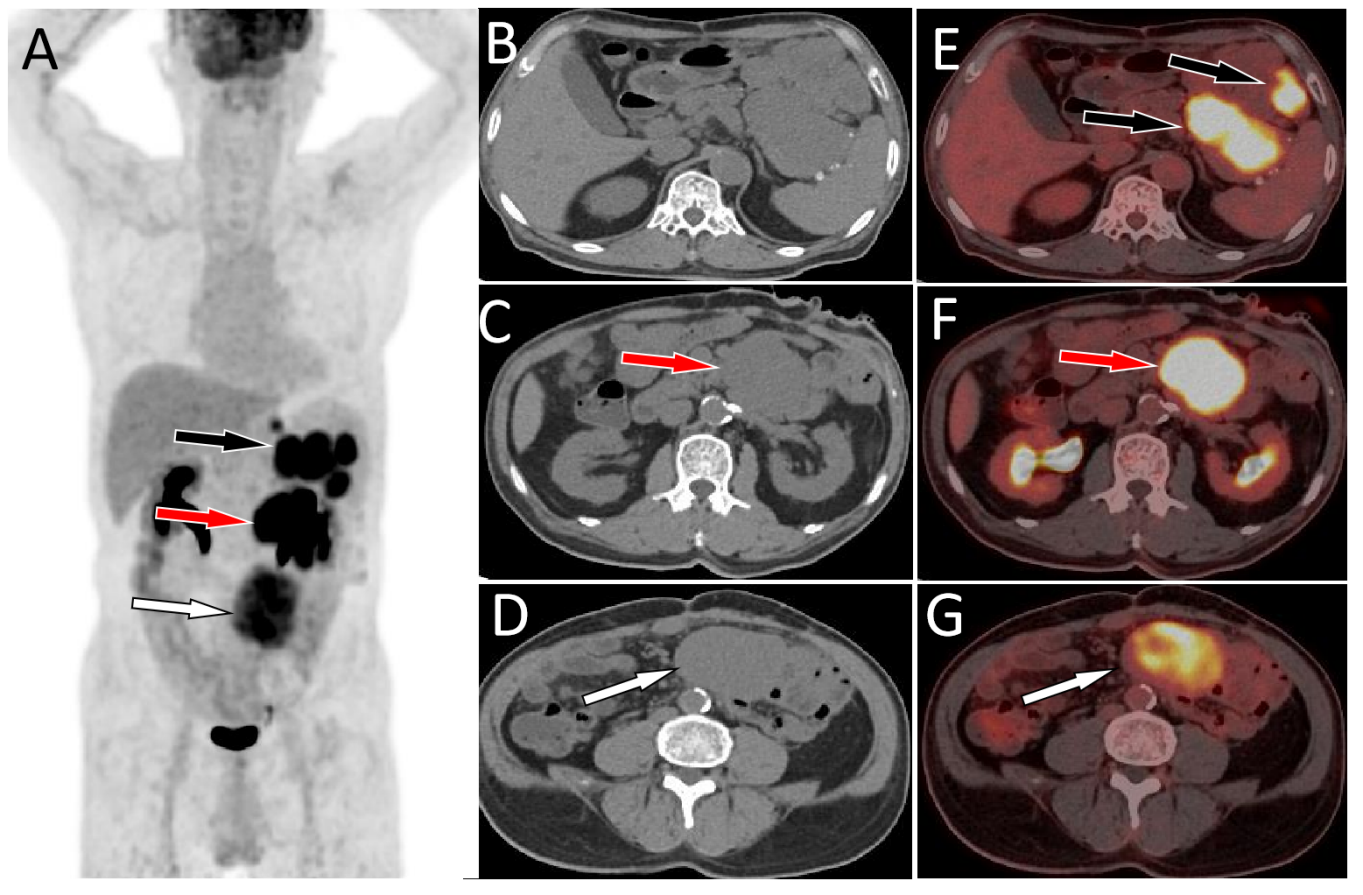
MIP image **(A)** acquired 60 min after injection showed that large intensity uptake in the left upper abdomen with SUVmax of 13.2 (black arrow), 14.6 (red arrow) and moderate radioactive uptake overlapping the spine with SUVmax of 9.1 (white arrow). On axial CT **(B-D)** and PET/CT fusion **(E-G)** images, the mass with high radioactivity uptake was located in the spleen-gastric space (black arrow), left retroperitoneum (red arrow), and right retroperitoneum (white arrow). Notes: MIP, maximum intensity projection; CT, computed tomography; PET, positron emission tomography; ^18^F-FDG, ^18^F-labeled fluoro-2-deoxyglucose; SUVmax, maximum standard uptake value.

**Figure 2:**
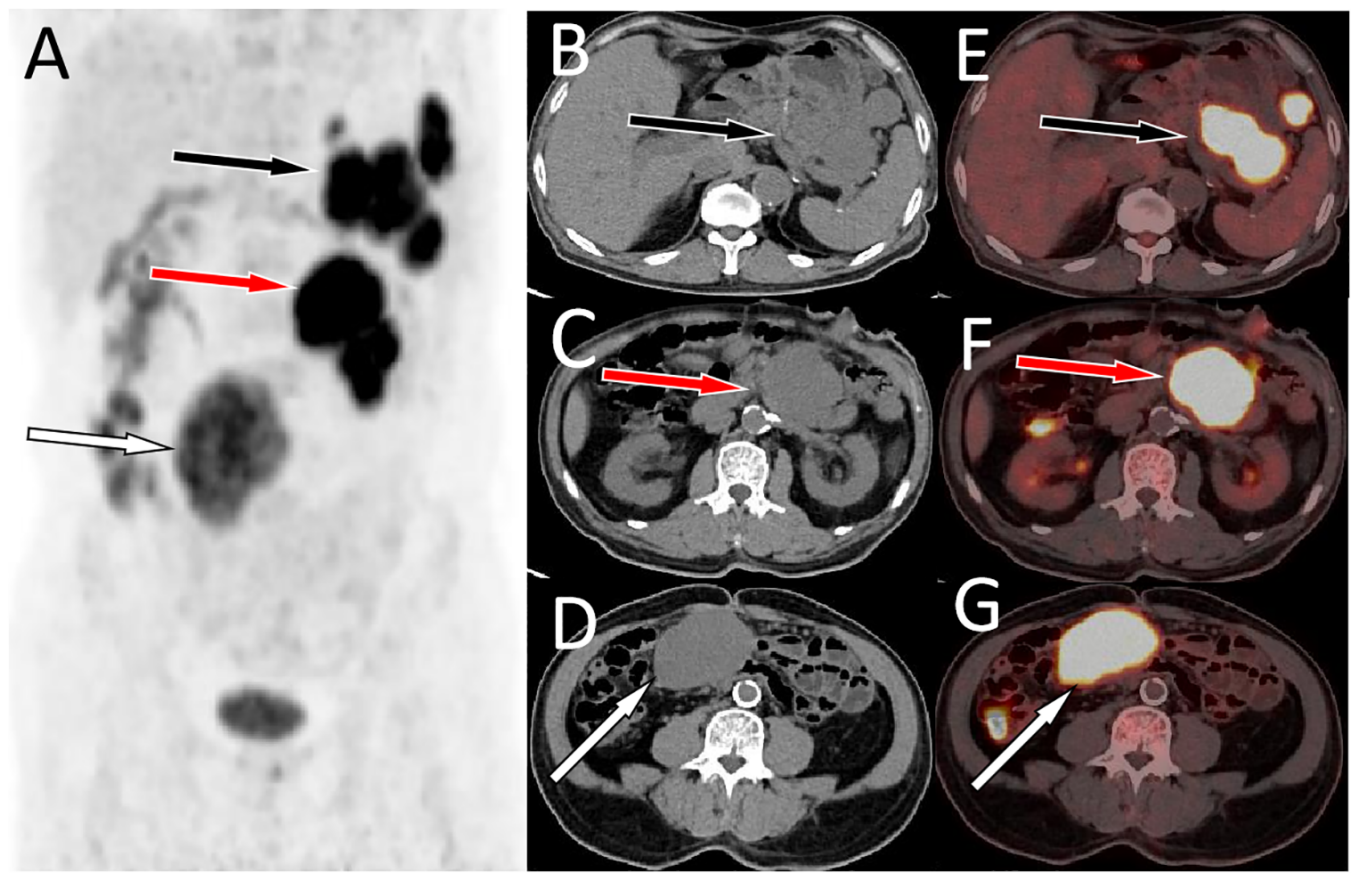
A delayed PET/CT scan at 180 min (**A**, MIP) was acquired, axial CT **(B-D)** and PET/CT fusion **(E-G)** showed a further increase in ^18^F-FDG radioactivity in the abovementioned lumps, with SUVmax of 16.0, 17.1, and 16.7 from top to bottom, respectively. Furthermore, the location of the lowest mass shifted from the initial left side of the spine to the right side of the spine (**G**, **I**, white arrows).Notes: MIP, maximum intensity projection; CT, computed tomography; PET, positron emission tomography; ^18^F-FDG, ^18^F-labeled fluoro-2-deoxyglucose; SUVmax, maximum standard uptake value.

**Figure 3:**
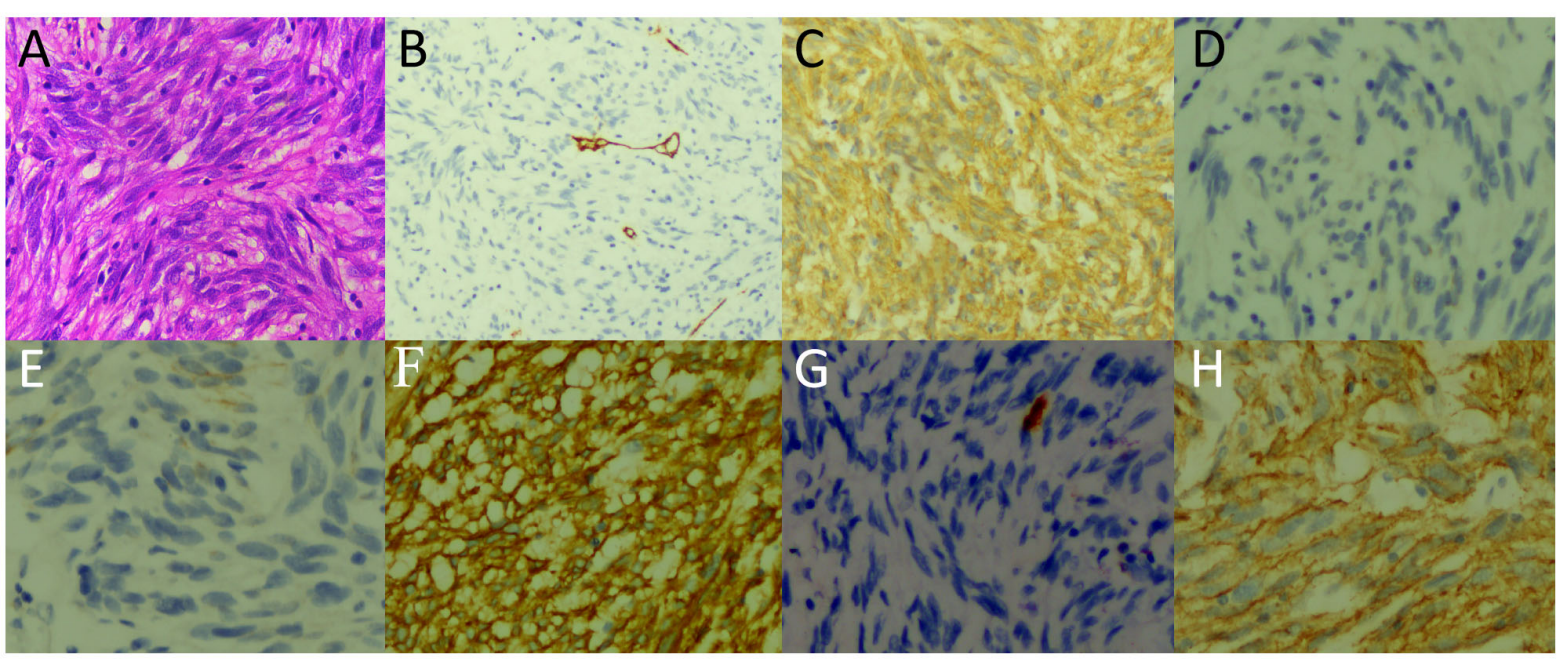
Hematoxylin and eosin staining showed uniformly dispersed fusiform cells **(A)**. Immunohistochemical results showed that the tumor expressed CD34 **(B)**, CD117 **(C)**, DESMIN **(D)**, CK **(E)**, DOG-1 **(F)**, S100 **(G)**, and SMA **(H)**.

A 64-year-old male patient suffered from recurrent abdominal pain and distension for 15 days who underwent upper abdominal CT scan in an external hospital 4 days ago and found abdominal mass. Now he came to our hospital for further diagnosis and treatment. Neither the patient nor his family had a history of tumors or genetic diseases. Routine physical examination revealed a slight elevation in serum Ca125 levels, with a value of 65.2 (reference value, <35). Other laboratory indicators and serum tumor marker values were within normal reference ranges. Whole abdominal CT revealed multiple soft tissue mass shadows in the abdominal cavity and retroperitoneum. Low-density cystic necrotic areas were observed in the mass. ^18^F-FDG PET/CT was recommended for the systematic evaluation of the patient’s condition. ^18^F-FDG PET/CT (Obtained after injection of ^18^F-FDG 60 min, **Figure 1**) revealed multiple soft tissue masses in the abdominal cavity and retroperitoneum, exhibiting heightened radioactive uptake, which were poorly demarcated from the surrounding structures, and the adjacent bowel was displaced by pressure (**Figure 2**). Furthermore, a delayed PET/CT scan at 180 min was performed to understand the delayed uptake of ^18^F-FDG by the mass over time and further determine the location of the mass and the relationship between the mass and the intestinal wall. The results indicated increased uptake of ^18^F-FDG in the mass, and presented migration of the mass in the lower abdomen, no longer retaining their initial position. Subsequently, the patient underwent exploratory laparoscopy, tumor resection, and complex intestinal adhesion lysis under general anesthesia. During the operation, it was found that the mass compressed the adjacent bowel without any direct connection. Therefore, it was considered that the mass may have originated from the mesentery. The lumps underwent pathological examination following the surgery. Hematoxylin and eosin staining revealed the presence of uniformly dispersed fusiform cells (as shown in **Figure 3**). Immunohistochemical results showed that the tumor positively expressed DOG-1, CD117, DESMIN, CK, CD34, S100, SMA, and vimentin, while it was negative for human melanoma black 45, CD56, NSE, and Bcl-2. Based on these findings, a diagnosis of GISTs (mitotic figure: 5-11/50 high power fields) was established. Subsequently, further genetic testing was conducted on these excised tumors, which revealed different KIT and PDGFRA mutation patterns, indicating a polyclonal origin. Consequently, the patient was diagnosed with multiple sporadic GISTs. After surgery, the patient underwent a 5-day anti-inflammatory treatment and was discharged without further chemotherapy. Subsequently, the patient underwent a 30-month follow-up, during which they remained healthy and alive.

## 3 Discussion

GIST are the most common stromal tumors in the gastrointestinal tract and can occur at various sites along this tract, with the stomach and small intestine being the most frequent locations. It is worth noting that certain GISTs may originate outside the gastrointestinal tract, including the omentum, mesentery, and retroperitoneum (10). Typically, GISTs occur as solitary entities, however, in rare instances, they may occur as multiple tumors distributed across one or more organs. Initially, the presence of multiple GISTs was primarily associated with familial GISTs, children, or specific tumor syndromes, such as NF1, Carney’s triad, and Carney-Stratakis syndrome (11). Furthermore, regardless of their number, size, or location, the occurrence of multiple GISTs is often considered tumor recurrence or metastasis. In 2007, Kang et al. examined five patients with sporadic multiple GISTs and identified different KIT gene mutation types (VVEEIN559-564del, V559, and V560 del) in distinct masses within the same patient. Each mass exhibited distinct cellular morphological and immunohistochemical characteristics, thereby proving the existence of sporadic multiple GISTs (7). Subsequently, Haller et al. conducted genetic testing on four patients with multiple GISTs and found that each tumor within the same patient had different KIT or PDGFRA mutation types, further confirming that these multiple GISTs were primary lesions (8). Multiple sporadic GISTs occurring outside of familial and symptomatic syndromes have been rarely documented and tend to occur in middle-aged and older adults, with a slightly higher incidence in men than in women (approximately 1.3:1) (12, 13). The most common clinical manifestations associated with multiple sporadic GISTs include abdominal pain, bloating, and intestinal obstruction resulting from compression of adjacent intestines by lumps (14). Here, we reported the case of a 64 year old male patient with no history of tumors or genetic diseases. The patient sought medical assistance for abdominal pain and bloating, which were consistent with the diagnosis and epidemiology of multiple sporadic GISTs.

The CT and magnetic resonance imaging (MRI) features of GIST, including large, circular, or lobulated soft tissue masses prone to cyst formation or necrosis in the center, and nonuniform delayed enhancement, have been reported in previous studies (15–17). Compared with traditional imaging techniques, PET/CT offers distinct advantages as it can simultaneously provide anatomical and metabolic information of lesions. Additionally, ^18^F-FDG PET/CT has been widely used for staging, re-staging, and monitoring the treatment response of GISTs (18, 19). The ^18^F-FDG PET/CT findings of GISTs correlate with their risk grade, with high-risk GISTs exhibiting a higher maximum standard uptake value (SUVmax) than that of medium-to low-risk GISTs (20). Furthermore, a previous study demonstrated a positive correlation between the SUVmax of ^18^F-FDG PET/CT and the Ki-67 index, tumor size and mitotic image. The sensitivity and specificity of SUVmax in predicting the risk grade were 85.7% and 94.7%, respectively. However, due to the rarity of multiple sporadic GISTs, the PET/CT findings have been less comprehensively documented in the literature. The case presented here showed multiple abdominal masses with unclear boundaries between adjacent structures. PET/CT revealed multiple lesions with varying degrees of uneven metabolic abnormalities in the abdomen and pelvis.

Multiple sporadic GISTs are rare, which should be differentiated from metastatic tumors, Castleman disease (CD), inflammatory myofibroblast tumor (IMT), and lymphoma (21). Metastases have a history of primary disease and varying degrees of radioactive uptake on PET/CT, according to the characteristics of the primary tumor. Most primary tumors can be visualized using PET/CT. The biological behaviors of IMT and CD are typically benign or tend to exhibit benign traits, featuring imaging characteristics akin to those of benign tumors, often presenting spherical soft tissue shadows with clear boundaries. The maximum standard uptake value of these tumors in PET/CT imaging is significantly lower compared to that of GISTs (22–24). Lymphoma, characterized by relatively uniform mass density and unclear boundaries with the adjacent gastrointestinal wall, is rarely necrotic and is often accompanied by multiple lymph node enlargements in the small and large curvatures of the stomach, abdominal trunk, or mesentery. Floating vascular shadows can be seen within the lesion, with a high uptake of ^18^F-FDG and an SUVmax >20 (25).

**Figure 4:**
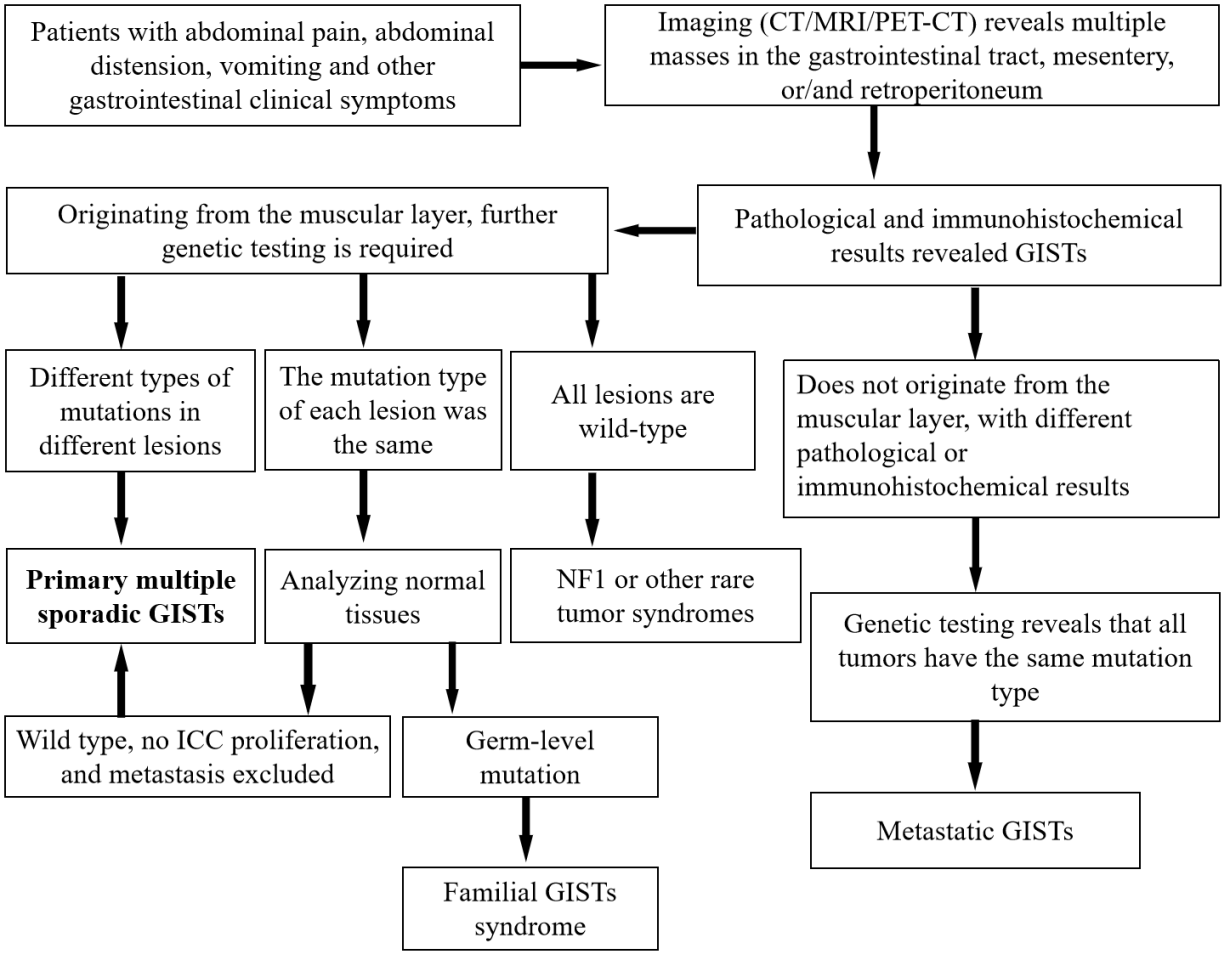
General diagnostic flowchart for multiple GISTs. CT, computed tomography; GISTs, gastrointestinal stromal tumors; ICC, interstitial cells of Cajal; MRI, magnetic resonance imaging; PET, positron emission tomography.

According to tumor cell morphology, GISTs can be divided into spindle, epithelioid, and mixed types (11). Distinguishing GISTs from other gastrointestinal tumors, such as non-GIST sarcomas and sarcomatoid carcinomas, based solely on tumor cytology can be challenging. Immunohistochemical testing can be used to diagnose suspected GISTs, among which CD117 and DOG-1 are the most important immunohistochemical detection indicators with high sensitivity and specificity. Moreover, the vast majority of GISTs positively express CD117 (95%) and DOG-l (98%) (26, 27). In addition to meeting the histopathological criteria for GISTs, the diagnosis of multiple sporadic GISTs also requires excluding familial GISTs, pediatric cases, or specific tumor syndromes such as NFl, Carney syndrome, and Carney-Stratakis syndrome. In the present case, spindle-shaped tumor cells were observed on microscopic examination, whose immunohistochemistry showed positive expression of CD117 and DOG-l; the patient had no evidence of familial GISTs or tumor syndromes. This aligns with the diagnosis of multiple sporadic GISTs. From a clinical perspective, diagnosing primary multiple sporadic GISTs is often more challenging than diagnosing GISTs accompanied by related tumor syndromes or metastasized disseminated GISTs. Nevertheless, accurate diagnosis significantly impacts disease staging and consequently influences treatment strategies. Therefore, to reduce the occurrence of clinical misdiagnosis, we have developed a diagnostic flowchart for GISTs, as shown in **Figure 4**.

Surgical resection is the preferred treatment for multiple sporadic GISTs, and the scope of surgical resection is determined based on the site, size, nature of the tumor, and the patient’s overall systemic condition (28). In patients with advanced stages or metastasis, imatinib can be used to alleviate the progression of the condition (11). Multiple sporadic GISTs can exhibit varying malignant potentials, ranging from benign to lethal malignant tumors. As a result, their prognosis is comparable to that of isolated GISTs. A previous extensive clinical study showed that 5-year and 15-year disease-free survival rates of GISTs were 70.5% and 59.9%, respectively (29). Our patient underwent tumor removal surgery without any additional treatments. After a 30-month follow up, the patient remains in good health and is still alive.

## 4 Conclusion

In conclusion, our study has shed light on the important role of PET/CT in the diagnosis of multiple sporadic GISTs, especially those originating outside the gastrointestinal tract. The migration of the mass in the lower abdomen was observed on dual time point PET/CT, and an increase in radiation uptake was observed 3 hours later compared to 1 hour after ^18^F-FDG injection, which is an important diagnostic indicator. This finding holds promising clinical implications, potentially influencing the early diagnosis and management of patients with GIST. The migration of the mass in the lower abdomen and differential uptake in dual-time-point PET/CT could provide valuable findings for healthcare professionals in making more accurate and timely diagnostic decisions.

## Data availability statement

The original contributions presented in the study are included in the article/supplementary material. Further inquiries can be directed to the corresponding authors.

## Ethics statement

Ethical review and approval was not required for the study on human participants in accordance with the local legislation and institutional requirements. Written informed consent from the [patients/participants OR patients/participants legal guardian/next of kin] was not required to participate in this study in accordance with the national legislation and the institutional requirements. Written informed consent was obtained from the participant/patient(s) for the publication of this case report.

## Author contributions

CL: Conceptualization, Data curation, Validation, Writing – original draft. WL: Methodology, Software, Validation, Writing – original draft. MS: Investigation, Project administration, Supervision, Writing – original draft. PW: Investigation, Supervision, Validation, Writing – review & editing. XH: Conceptualization, Formal analysis, Methodology, Resources, Writing – review & editing.
